# Southern Dental Association

**Published:** 1884-09

**Authors:** 


### SOUTHERN DENTAL ASSOCIATION.

SIXTEENTH ANNUAL MEETING, HELD AT LEXINGTON, KY., MAY 6, 7, 8 AND 9, 1884.

REPORTED EXPRESSLY FOR THE INDEPENDENT PRACTITIONER.

(Concluded from page 448.)

### AFTERNOON SESSION.

*Dr. A. O. Rawls*—Desired to call attention to that bugbear—so called, because it is not understood—the etiology, pathology, and means of cure of the disease *pyorrhœa alveolaris.* He (Dr. Rawls) had stated his views, and had the support of Dr. Rehwinkel and several others ; that the disease is constitutional with a local indication. It invariably follows the use of mercury. It is transmitted from parent to child, even to the third generation. It is of the same nature as scurvy, but is not the same in the general system. Similar conditions are found where tomatoes are largely used; canned tomatoes especially. It is well known that mercury acts upon the liver, and that tomatoes act exactly in the same way, though with less effective results. It will be found that with tomatoes the same results can be produced as with mercury. He knew of but one common constituent in salt, mercury (calomel?) and tomatoes, that is: chlorine; therefore he concluded there must be some combination into which it entered to produce an influence on these peculiar tissues. The only way of cure, he fancied, was to remove all sources of irritation; not only remove the tartar, but cut away the diseased tissue. After the operator had done all he could with his instruments, he would be simply waiting for the disease to take hold of the other parts. He had heard of different medicaments, of antiseptics; but they do not radically cure the disease. They only stop the further action of the irritants.

*Dr. Taft*—Was somewhat in doubt as to the heredity of mercurial taint. When one becomes deteriorated, the progeny will not be strong; but he doubted whether a positive mark could be transmitted.

*Dr. Rawls*—Held that if we believe in the transmission of a single physical quality, we must believe in the transmission of mercurial taint. Consumption is hereditary; scrofula is hereditary. In these the transmitted molecules are similar to those of the tissues of the parent. We do not know that the mercury is carried along; we have no right to believe that; but we have a right to believe that its effect upon the tissues is transmitted in accordance with the peculiar surroundings of parent and child.

*Prof. Taft*—Admitted the transmission of constitutional peculiarities, at least those of static character; but those of an accidental nature are not transmitted, such as the loss of a finger, or the loss of part of a tissue. If inherent, they will, but if accidental, they will not be transmitted. The mercurial condition is only accidental.

*Dr. F. Peabody*—Agreed with Dr. Rawls about the transmission of the disease. He had seen in mother, son and daughter, the disease in the same tooth. The theory as to mercury might be correct; but, if so, it was more from the dying-out effects than the positive effects. Much mercury was given years ago, all through this southern country. Then the disease was not known; but now we see much of it. He believed it was a systemic or "blood" disease.

*Dr. Patrick*—Stated his experience to be that when the disease had not progressed too far, cleanliness was the best thing. He considered it local, and not constitutional, though the anatomical form of the teeth and alveola might be hereditary, and this makes certain teeth more liable to dislodgement. All the successful treatment he had heard of had been local. Those treating the disease called it a constitutional affection, but treated it locally. The transmission of disease was questionable in any case. There were some conditions of body in which more lime was secreted than in others. Thus far this disease might be constitutional in the deposit of tartar. He had never found satisfactory proof that it was in the blood.

*Prof. Smith*—Considered that peroxide of hydrogen was not the thing to use in such a disease. Putrefaction requires free oxygen. The bacteria feed on pure oxygen. He agreed that cleanliness was the best remedy. The germ might begin in the infant when nursing, as from a filthy nipple.

*Dr. A. Berry*—Said this was a common disease, now and formerly. It occurred in persons otherwise healthy, and was too often neglected. If the patient had not the fortitude to stand heroic treatment, he had better be let alone.

*Prof. Smith*—Desired to speak further on nursing. Since he had taken his seat a book had been placed in his hands. It was the INDEPENDENT PRACTITIONER. In it he found a paper by Dr. Miller, of Berlin, Germany, on fermentation in relation to caries of the teeth, in which lactic acid was referred to as an agent. Now, if lactic acid ferment be a cause of caries, why not of *alveolaris pyorrhœa?* His attention had been first called to the probability of disease arising in the mouth of an infant by seeing a very filthy mother suckling a child. It was well known that many mothers were very careless in regard to the cleanliness of the nipples in nursing, and this might easily be the origin of disease of the teeth.

THIRD DAY.

The Association visited "Ashland" in the morning, and being invited also to visit "Ashland Park," the farm of B. J. Tracy, an extensive horse-breeder, the members spent the entire forenoon at these two places. Failure to catch the train prevented the visit to Alexander's farm in the afternoon, but no session was held during the day.

EVENING SESSION.

A paper by Dr. B. H. Teague, of Aiken, S. C., on dental literature, was then read. After the transaction of considerable routine and local business the Association proceeded to the election of officers for the ensuing year, which resulted as follows:

President, Dr. A. O. Rawls, Lexington, Ky.; 1st Vice-President, W. R. Clifton, Waco, Texas; 2d Vice-President, T. S. Waters, Baltimore, Md.; 3d Vice-President, B. H. Catching, Atlanta, Ga.; Corresponding Secretary, J. L. Fountain, Bryan, Texas; Recording Secretary, R. A. Holliday, Atlanta, Ga.; Treasurer (re-elected), H. A. Lawrance, Athens, Ga.

THE NEXT MEETING.

On motion it was decided to hold the next annual meeting in New Orleans, on the last Tuesday in March, 1885, to which time the convention adjourned.

